# Case Report: Frostbite in the tropics: abdominal pedicled flap reconstruction to prevent the metacarpal hand

**DOI:** 10.3389/fsurg.2026.1829574

**Published:** 2026-05-15

**Authors:** Adzim Poh Yuen Wen, Zhun Shen Tan, Kah Ken Kwan, Mohamed Nabil Mohamed Nazri, Shalimar Abdullah

**Affiliations:** 1 Plastic Surgery Unit, Department of Surgery, Hospital Canselor Tuanku Muhriz UKM, Kuala Lumpur, Malaysia; 2 Faculty of Medicine, National University of Malaysia, Kuala Lumpur, Malaysia; 3 Hand & Microsurgery Unit, Department of Orthopaedics and Traumatology, Hospital Canselor Tuanku Muhriz UKM, Kuala Lumpur, Malaysia

**Keywords:** abdominal pedicled flap, case report, digital salvage, frostbite, hand reconstruction, hyperbaric oxygen therapy, metacarpal hand, prosthesis

## Abstract

The reconstructive salvage of a severely frostbitten hand in a tropical setting presents a unique challenge. We report a 59-year-old male who sustained extensive frostbite to the face, ears and bilateral hands following a Mount Everest ascent. Hyperbaric oxygen therapy (HBOT) was initiated during demarcation to optimise tissue viability. Following demarcation, dry gangrene of the right hand required amputation, revealing viable 1.5–2 cm proximal phalangeal stumps in all five digits. To avoid a non-functional metacarpal hand, reconstruction aimed to preserve digital length and metacarpophalangeal joints. A staged abdominal pedicled flap was performed, with initial flap inset and delayed pedicle division at 3 weeks, followed by syndactyly release. At 10-month follow-up, secondary debulking and contracture release were performed to optimise prosthetic use. The patient achieved stable, partially sensate digital stumps with preserved length and joint motion. This case highlights a two-stage reconstructive approach integrating HBOT and an abdominal pedicled flap to maximise anatomical preservation and functional outcome in severe pan-digital frostbite.

## 1 Introduction

Frostbite is a thrombotic ischemic injury from tissue freezing, involving immediate cellular cryoinjury and progressive dermal ischemia mediated by inflammatory cascades and microvascular thrombosis (1, 2). Classically associated with cold climates, frostbite presents a unique clinical challenge in tropical regions almost exclusively linked to high altitude mountaineering (1–3). For clinicians there, it remains a rare entity with potentially devastating functional and psychological consequences, particularly for the hand (2–6).

The hand is especially vulnerable due to its superficial anatomy, as severe frostbite frequently leads to digital necrosis necessitating amputation (1–3, 6). Functional deterioration in terms of hand function and grip strength is progressive with each amputated digit, most severe when the index is lost alongside digits three through five (5). The metacarpal hand, a hand that has lost all digits proximal to the functional length (mid-proximal phalanx) represents a severely debilitating endpoint that lacks basic prehensile function, capacity for opposition (6, 7).

Therefore, the reconstructive goal in severe digital frostbite shifts from simple wound closure to strategic anatomical preservation (2, 3, 7). The imperative is salvaging bone length to prevent this deformity, as preserving even a short proximal phalanx maintains the metacarpophalangeal (MCP) joint and intrinsic muscle insertions, offering superior functional prognosis (3–5, 7). This report details a case of severe pan-digital frostbite where this principle guided a two stage salvage using an abdominal pedicled flap and hyperbaric oxygen therapy (HBOT) to achieve simultaneous pan-digital coverage and functional residual length preservation (1, 3).

## 2 Case report

A timeline summarizing the key clinical events is provided in Table 1. A 59-year-old male with no known medical illnesses presented with severe frostbite to his face, ears, and bilateral hands following a successful Mount Everest summit in May 2023. A hypoxic event during descent led to prolonged, unprotected exposure. Evacuated to Kathmandu, Nepal, he received initial management including thrombolysis with alteplase for digital artery occlusion identified on angiography.

**Table 1:** Timeline of clinical course and reconstructive management.

Time from presentation	Clinical event
0 months	Initial presentation with extensive frostbite involving face, ears, and bilateral hands
1 month	Completion of 10 sessions of HBOT, noted clear demarcation of dry gangrene affecting all digits
1.2 months	1st Stage: Guillotine amputation with preservation of 1.5–2 cm proximal phalangeal stumps and immediate abdominal pedicled flap inset
1.8 months	2nd Stage: Pedicle division, flap inset, split-thickness skin grafts (STSGs) to volar surfaces and donor site, abdominal wound refashioning
4 months	Interval syndactyly release with STSGs for webspace reconstruction
5 months	Secondary abdominal scar revision with abdominoplasty
6 months	Final syndactyly release achieved separation into stable, partially sensate digital stumps with preserved length and joint motion.
10 months	Debulking and contracture release to optimise prosthetic fitting
Outcome	Functional preservation of digital stumps with prosthetic integration

**Figure 1:**
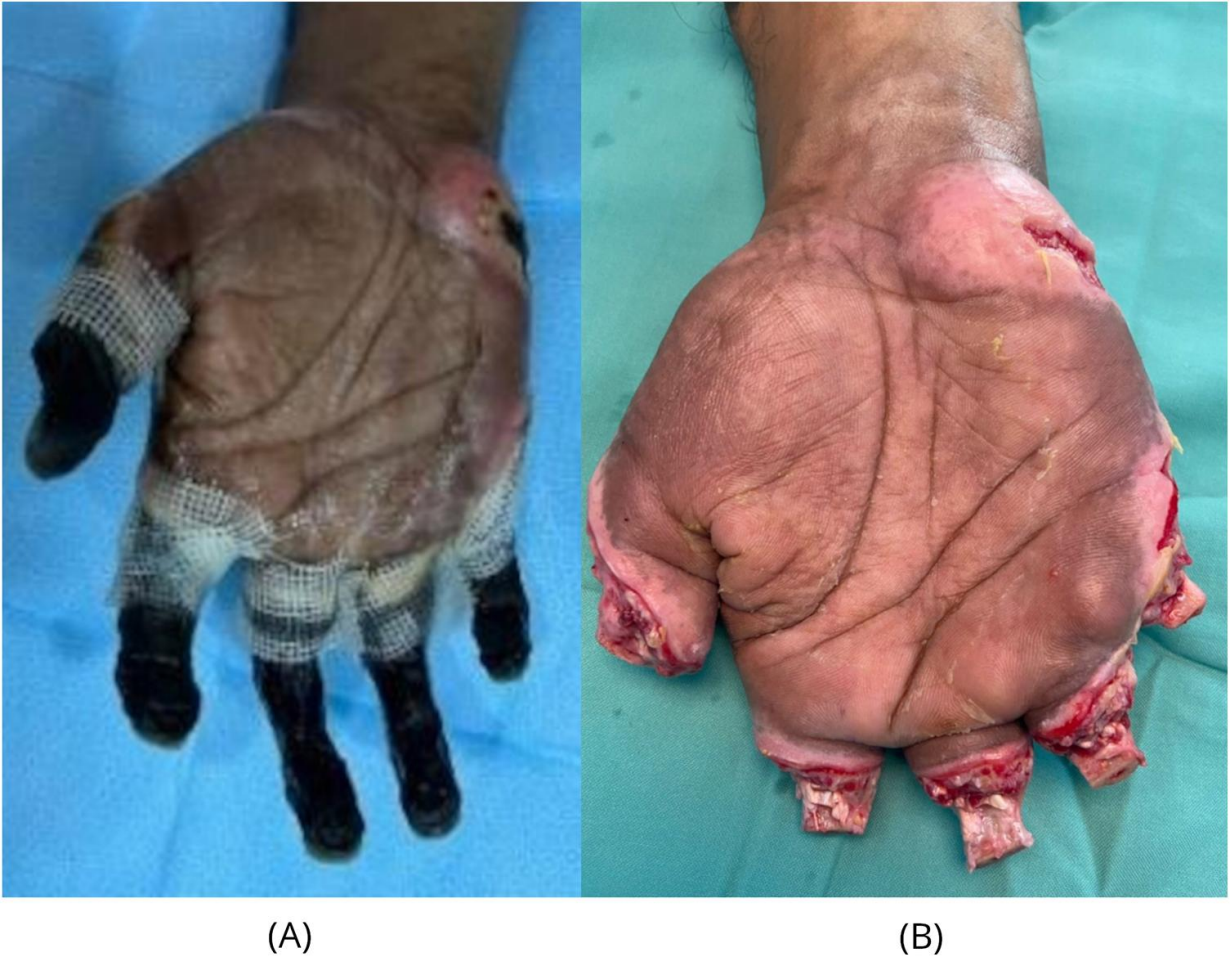
Demarcation and intraoperative findings of the right hand following frostbite injury. **(A)** Clinical appearance following demarcation, demonstrating dry gangrene affecting all five digits. **(B)** Intraoperative findings post-amputation, revealing preservation of 1.5–2 cm of viable proximal phalangeal bone in all digits.

Repatriated to Malaysia, a multidisciplinary team was convened and adjuvant HBOT was commenced preoperatively to optimize tissue viability during the demarcation phase. Following 10 sessions of 90 min HBOT, clear demarcation of dry gangrene developed in the right hand (Figure 1A). In contrast, the left hand was managed conservatively with HBOT alone, with preservation of tissue viability and no progression to necrosis. No surgical intervention was required and the hand remained largely unaffected with a near-normal clinical appearance.

Surgical exploration confirmed necrosis extending to the base of the proximal phalanx in the 4 fingers and the interphalangeal joint of the thumb. No identifiable digital nerve structures were visualised intraoperatively. Therefore, formal nerve repair was not performed. 1.5–2 cm of viable, bleeding proximal phalangeal bone was preserved in all 5 digits with residual tendon attachments to the stump, contributing to functional movement (Figure 1B).

**Figure 2:**
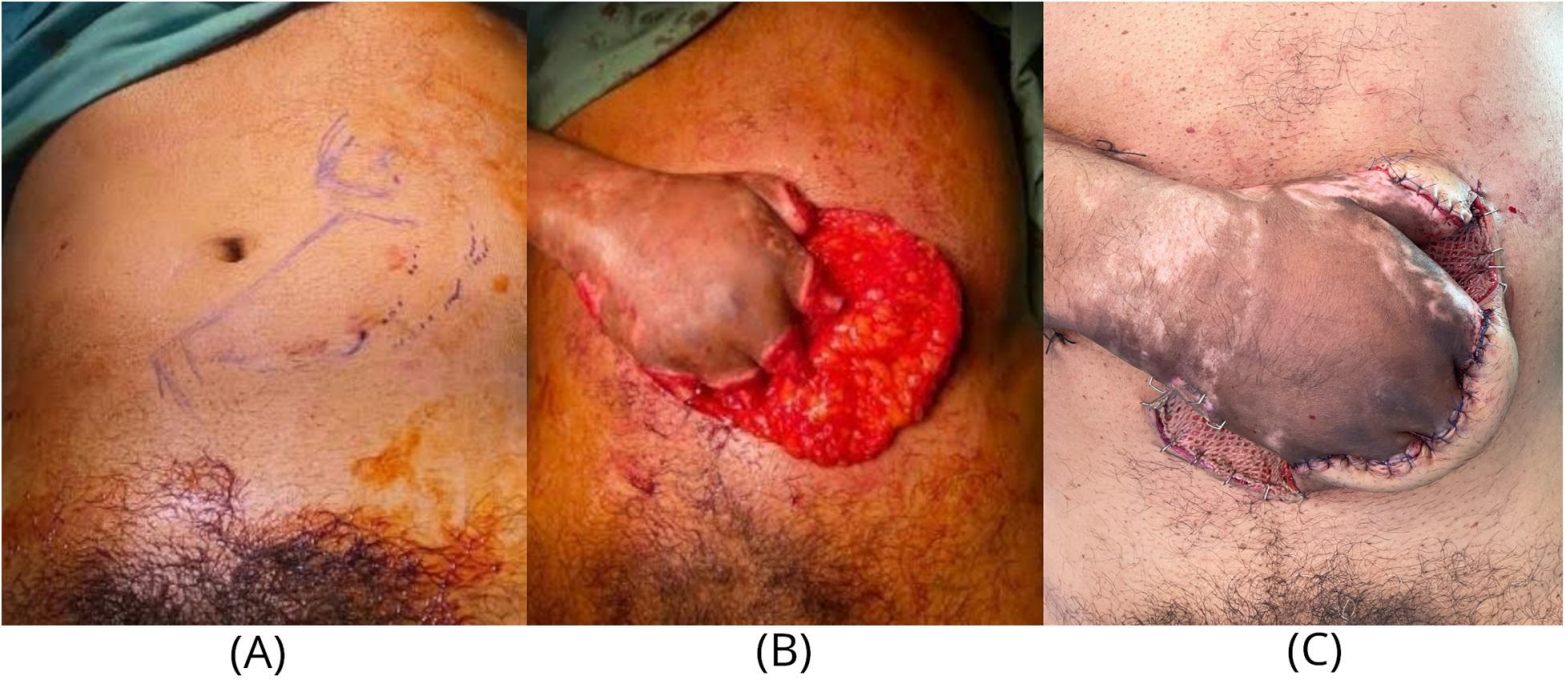
First-stage reconstruction with abdominal pedicled flap. **(A)** Flap design over the lower abdomen with a 2:1 length-to-width ratio. **(B)** Elevation of the abdominal flap at the pre-Scarpa's plane preserving subdermal vascularity. **(C)** Immediate postoperative inset showing the hand immobilized against the abdomen, forming a vascularized soft tissue “mitten”.

An aggressive salvage strategy was chosen over a higher-level metacarpal hand amputation. The first stage involved guillotine amputation at the level of viable bone and immediate inset of the abdominal flap to envelop all 5 stumps. The abdominal pedicled flap was designed with a length-to-width ratio of 2:1 to ensure flap viability (Figure 2A). It is a random pattern flap based on the subdermal and intradermal plexus, elevated at the pre-Scarpa's plane to preserve adequate subdermal vascularity (Figure 2B). The pedicle was maintained to ensure reliable perfusion throughout the period of inset where donor site closure was performed in layers which included the Scarpa's fascia, dermis and skin. This created a vascularized soft tissue “mitten” immobilized against the abdomen (Figure 2C). The flap underwent a 3-week delay to ensure robust neovascularization from the hand, augmented by serial, partial flap releases under local anesthesia beginning in week 2.

**Figure 3:**
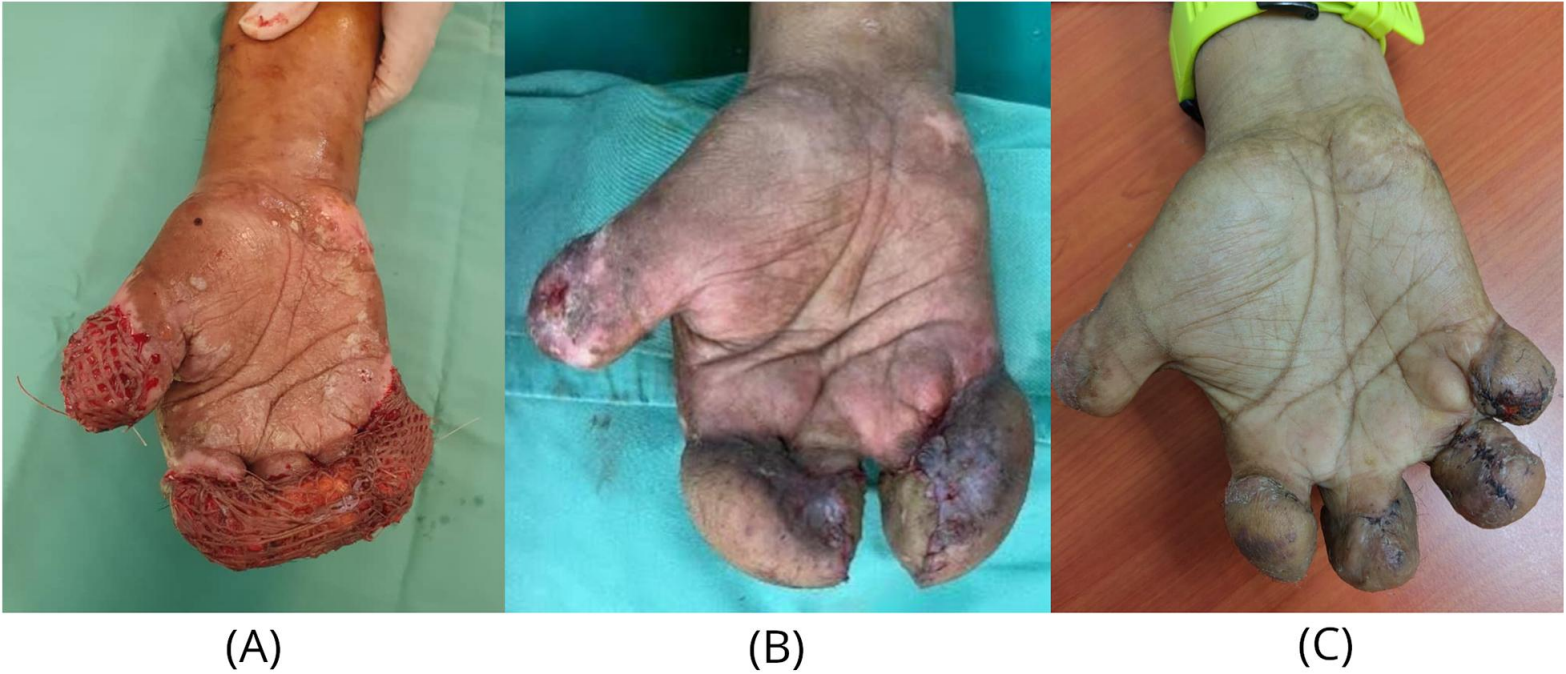
Second-stage reconstruction and intermediate outcomes. **(A)** Post-pedicle division with supplementary split-thickness skin grafting to the volar adipofascial surfaces. **(B)** Interval syndactyly release procedures to separate digital stumps and reconstruct webspaces. **(C)** Results at 10-month postoperative follow-up, showing preservation of all digital stumps which were debulked to facilitate prosthetic fitting and wear.

The second stage involved division of the pedicle, flap tailoring and definitive inset. During the inset, there was an underestimation of required tissue preservation leading to over-excision of subcutaneous tissue. As primary flap coverage was not feasible, this necessitated supplementary coverage of the volar adipofascial surfaces of the flap with split-thickness skin grafts which were used to resurface the abdominal donor site as well (Figure 3A).

The postoperative course was uncomplicated. Subsequent syndactyly releases were performed at 2-month intervals to separate the stumps and reconstruct the webspaces (Figure 3B). At 4-months, the patient demonstrated stable, partially sensate digital stumps with preserved MCP joint motion, successfully preventing a metacarpal hand. Sensibility was not formally assessed. Subjectively, the patient reported approximately 30% deep pressure sensation at the stumps compared to adjacent normal areas. Bulky contours and limited digital motion prompted plans for debulking and contracture release at 10-months to optimize the platform for prosthetics (Figure 3C) (Supplementary Video S1).

The patient expressed satisfaction with functional hand preservation with prosthetic potential despite later concerns regarding donor-site scarring, for which he consented to secondary revision. Functionally, he was able to perform basic activities of daily living using the prosthesis, including writing with a pen and unscrewing objects. However, more detailed functional assessment and video documentation of task-specific performance were not obtained.

## 3 Discussion

This case highlights a reconstructive strategy in severe frostbite where anatomical preservation is prioritized to avoid the functionally devastating metacarpal hand (7). Viable proximal phalangeal bases presented a critical crossroad between accepting a higher amputation level for simpler healing or undertaking complex reconstruction to preserve functional architecture (3). The latter was chosen with the principle that preserving the MCP joint and intrinsic muscle insertions provides significant functional improvement compared to a metacarpal hand (5, 7).

In managing such complex injuries, selection of the reconstructive modality is critical (8, 9). In frostbite, diffuse microvascular thrombosis compromises recipient vessel quality, rendering free tissue transfer less reliable and increasing the risk of partial or complete flap loss (1, 2, 8). This risk is particularly unacceptable when simultaneous salvage of all 5 digital stumps is required (2). In contrast, pedicled abdominal flaps offer a technically reliable alternative in the setting of compromised distal vasculature, shorter operative times and lower complication rates (2, 3, 8). Their large surface area enables simultaneous pan-digital coverage without the need for temporary syndactylization (3). In this case, an abdominal pedicled flap was therefore selected as the workhorse reconstructive option, with a planned 3-week delay to enhance neovascularization and ensure flap viability prior to pedicle division (1, 3).

While the groin flap is traditionally considered a workhorse option for hand reconstruction (3), several factors influenced the decision to utilise an abdominal pedicled flap in this case. In our local setting, the abdominal pedicled flap is often preferred due to its ability to provide greater and more reliable tissue bulk (3), which may be influenced by anthropometric variation in soft tissue distribution across different populations. Previous studies on body composition and flap characteristics have demonstrated variability in subcutaneous tissue thickness between ethnic groups and anatomical regions (10), allowing cautious extrapolation that groin flap bulk may be less predictable in certain populations. However, this remains an area requiring further dedicated investigation. This is especially important when simultaneous coverage of multiple digital stumps is required. Additionally, the period of immobilisation during groin flap inset may result in significant patient discomfort (3), with reduced tolerance observed beyond the first week in our institutional experience. In contrast, the lower abdominal region offers a more generous and reliable source of tissue while allowing for improved patient comfort during immobilisation. Furthermore, the abdominal donor site shares anatomical and technical similarities with standard abdominoplasty, with comparable complication profiles reported in the literature, supporting its acceptability as a donor site in reconstructive procedures (11). These factors collectively supported the selection of an abdominal pedicled flap over alternative reconstructive options in this case.

**Figure 4:**
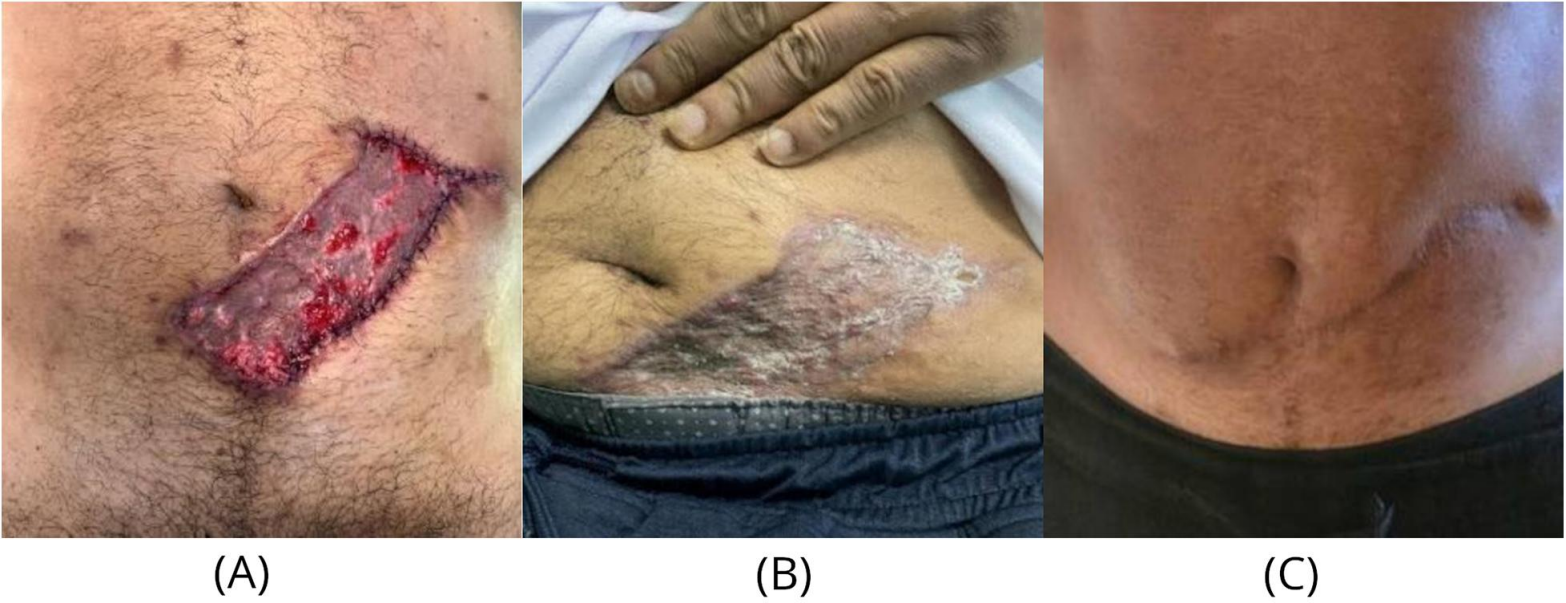
Donor-site outcome and secondary revision. **(A)** Abdominal donor-site appearance after Stage 2 with split-thickness skin graft (STSG) inset. **(B)** Interval abdominal donor-site scar prior to abdominoplasty with patient-reported aesthetic concern. **(C)** Post–scar revision and abdominoplasty demonstrating improved contour and scar appearance.

Donor-site morbidity remains an important consideration in abdominal pedicled flap reconstruction where the resulting abdominal scar may have aesthetic implications and impact patient satisfaction (8). In this case, the patient later expressed concern regarding the donor-site appearance (Figure 4B) and subsequently underwent secondary scar revision combined with abdominoplasty approximately 5 months postoperatively (Figure 4C). This highlights the need for preoperative counselling regarding potential donor-site outcomes and the possibility of secondary procedures.

HBOT was employed as a preoperative adjunct during the demarcation phase (3). While not being a standard protocol in frostbite management, HBOT offers several physiological benefits in ischemic conditions (1). It increases tissue oxygen tension, reduces edema, modulates inflammation and promotes angiogenesis (1). HBOT has been shown to increase the probability of limb or segment preservation by mitigating inflammatory cascade activation and ischemia-reperfusion injury (1). The excellent wound bed quality and robust bleeding observed during flap division may be partially attributable to this adjunctive therapy, supporting a potential role for HBOT in optimizing frostbite wounds prior to reconstruction (1).

Comparative data between HBOT and non-HBOT cohorts in both abdominal and groin flap reconstruction remain limited. However, existing evidence from studies on mutilated hand injuries demonstrates that adjunctive HBOT can improve survival of compromised tissues and flaps, with reported survival rates of up to 81% in replanted digits and 100% in palmar tissues following reconstruction (12). These findings suggest that the benefits of HBOT are not specific to flap type, but rather related to optimisation of the wound environment and preservation of the zone of stasis. In this context, HBOT may serve as a valuable adjunct across reconstructive strategies including abdominal and groin flaps by improving tissue viability prior to definitive coverage. However, given the heterogeneity of injuries and lack of direct comparative studies, its role remains supportive rather than definitive and further studies are required to establish its impact on flap-specific outcomes.

The use of STSGs on the volar aspect is not routine in hand reconstruction due to concerns regarding functionality and cosmetic results compared to full-thickness skin grafts (2). However, in this case, STSGs were necessitated by intraoperative limitations following underestimation of required tissue preservation. While representing a compromise from the ideal reconstructive option, this enabled complete coverage of exposed structures while preserving skeletal length and joint integrity, the primary objective of reconstruction (3). The requirement for secondary debulking and contracture release is anticipated in major reconstructions of this nature (2, 8). In selected cases, active exercises of the attached hand may be implemented during immobilization to reduce stiffness (9). The resulting separate digital stumps provide a markedly superior foundation for both biomechanical function and advanced bio-prosthetic integration compared with metacarpal amputation (3, 6).

In conclusion, severe frostbite demands a reconstructive philosophy centered on length preservation. This case demonstrates that a staged abdominal pedicled flap integrated with preoperative HBOT can simultaneously salvage multiple digital stumps, preventing the functionally limiting metacarpal hand deformity. This reliable technique remains an essential component of the reconstructive ladder for complex upper-extremity salvage, particularly when microvascular anastomosis is contraindicated by compromised vessel quality.

## 4 Limitations

This case report has several limitations. As a single case report, findings cannot be generalised and causality cannot be established. The absence of a formal patient-reported outcome measure limits objective quantification of functional recovery and quality of life following reconstruction. Additionally, the fifth digit could not be fitted with a prosthesis due to insufficient residual stump length representing an incomplete functional outcome.

Long-term follow-up beyond 10 months is not yet available and future documentation will be needed to confirm the durability of reconstruction and sustained prosthetic integration. Detailed functional video documentation demonstrating task-specific prosthetic use was not available. Objective sensory assessment using standardized tools was also not performed. Future cases will incorporate structured functional assessments and validated sensory testing to better quantify outcomes.

## Data Availability

The original contributions presented in the study are included in the article/Supplementary Material, further inquiries can be directed to the corresponding authors.

## Supplementary material

Supplementary Video S1 This video demonstrates the postoperative functional outcome with MCPDriver prostheses (Naked Prosthetics, Inc., Olympia, WA, USA) following digital salvage and reconstruction. The fifth digit was not fitted due to insufficient residual stump length. The patient reported occasional nonuse of the thumb prosthesis because of discomfort but maintained functional thumb use due to adequate stump length.
